# Editorial: Spread of pest and diseases of pollinators in a changing world

**DOI:** 10.3389/finsc.2026.2014558

**Published:** 2026-10-22

**Authors:** Chuleui Jung, Terd Disayathanoowat

**Affiliations:** 1 Department of Plant Medicals, Gyeongkuk National University, Andong, Republic of Korea; 2 Agricultural Research Institute, Gyeongkuk National University, Andong, Republic of Korea; 3 SMART BEE SGDs Research Center, Department of Biology, Faculty of Science, Chiang Mai University, Chiang Mai, Thailand

**Keywords:** detoxification, pesticide, Tropilaelaps, Varroa, virome

## Introduction

Pollinators are fundamental to the reproduction of wild plants and to the production of many crops contributing human nutrition and agricultural diversity (1, 2). Yet managed and wild pollinators are increasingly exposed to multiple and interacting stressors, including habitat degradation, nutritional limitation, pesticides, parasites, pathogens, invasive species, and climate change (3). These pressures rarely act independently. Climate change can modify host and parasite distributions and generate phenological mismatches between pollinators and flowering crops (4), while global trade and movement of managed bees facilitate the transboundary spread of parasites and pathogens. This Research Topic, “Spread of Pest and Diseases of Pollinators in a Changing World,” brings together six contributions that illustrate this complexity from parasite surveillance and viral diversity to molecular host responses, pesticide effects, and potential mitigation strategies (**Figure 1**).

**Figure 1:**
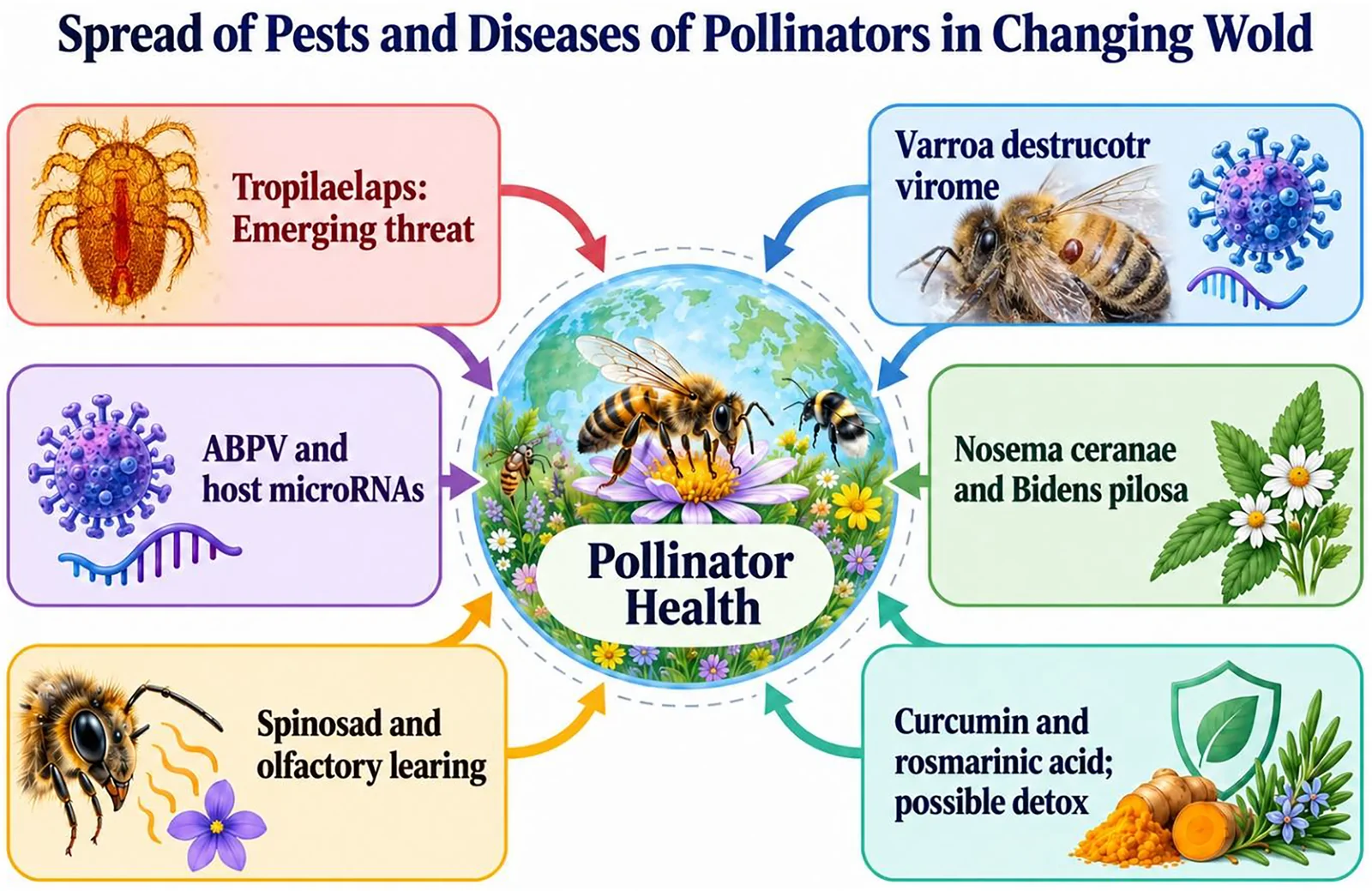
Graphical overview of the six contributions addressing pollinator health in a changing world. The Research Topic integrates six complementary themes related to major biological and chemical stressors affecting pollinators: the emerging threat of *Tropilaelaps*, the virome associated with *Varroa destructor*, ABPV–host microRNA interactions, *Nosema ceranae* responses to *Bidens pilosa*, sublethal effects of spinosad on olfactory learning, and the potential detoxifying effects of curcumin and rosmarinic acid. Together, these studies illustrate the need for an integrated understanding of parasites, pathogens, pesticides, host responses, and mitigation strategies in maintaining pollinator health under global environmental change. Photo of Tropilaelaps mite from Sunho Kwon and Chuleui Jung, and varroa on honey bee from Chuleui Jung.

A central theme is the continuing emergence and redistribution of parasitic mites. *Varroa destructor* remains one of the most important biological threats to *Apis mellifera*, both through direct parasitism and through its role in altering the transmission and epidemiology of honey bee viruses (5). Kim et al. provide a comprehensive virome analysis of *V. destructor* populations in South Korea, emphasizing that mite surveillance should extend beyond infestation intensity to the pathogens associated with mite populations. Such approaches are increasingly relevant because parasite prevalence and risk vary with management and landscape context, as demonstrated for *V. destructor* and *Tropilaelaps mercedesae* in Korean apiaries (6).

The geographical expansion of *T. mercedesae* deserves particular attention. Esmaeily et al. (2026) synthesize current knowledge of its biology, distribution, diagnosis, and management and identify this mite as an emerging global threat to apiculture. Its rapid reproductive cycle, dependence on honey bee brood, and potential for movement through managed colonies make early detection especially important. The growing concern surrounding *Tropilaelaps* illustrates how historically regional pests can become international biosecurity issues when climate suitability, bee movements, and trade interact. Standardized monitoring, improved diagnostic tools, international information exchange, and integrated control strategies will therefore be essential.

Pathogens represent another important dimension of pollinator health. Kumar et al. investigate acute bee paralysis virus infection in a honey bee-derived cell line and demonstrate changes in host microRNA expression following infection. By moving from pathogen detection toward regulatory mechanisms, their study highlights the potential role of microRNAs in antiviral defense and viral pathogenesis. Complementing this molecular perspective, Li et al. examine the effects of *Bidens pilosa* phytogenic treatment on *Nosema ceranae* gene expression in infected honey bees. Their results illustrate the growing potential of transcriptomic approaches to identify biological pathways affected by candidate phytogenic treatments and to provide mechanistic support for disease-management strategies.

Chemical stressors add another layer of complexity. Pollinators encounter pesticides used in crops as well as veterinary compounds applied within colonies, creating opportunities for mixture effects that conventional single-compound risk assessments may overlook. Species sensitivity also differs substantially; comparative testing of four Asian honey bee species showed that pesticide toxicity cannot necessarily be extrapolated from *A. mellifera* to other *Apis* species (7). Likewise, combined exposure to agricultural insecticides, fungicides, bactericides, or in-hive acaricides can alter toxicity. Recent studies have documented interactive risks involving copper hydroxide and co-occurring pesticides (8) and increased honey bee vulnerability when amitraz exposure occurs together with organophosphate insecticides (9).

Two contributions in this Research Topic address pesticide-related effects and possible mitigation. Rondoni et al. developed a microcontroller-based device to standardize proboscis extension reflex conditioning and showed that spinosad exposure can impair olfactory learning. This study reinforces the need to evaluate behavioral and other sublethal endpoints rather than relying solely on mortality. At the same time, Mohamadzade Namin et al. explored curcumin and rosmarinic acid as dietary tions against pesticide-induced harm. Their findings suggest that phytochemicals may enhance detoxification or stress resilience under some exposure conditions. Such approaches are promising, but laboratory benefits should ultimately be validated at colony and field scales and should complement, rather than replace, efforts to reduce hazardous exposure.

Taken together, the six contributions emphasize a common message: pollinator health is a multi-stressor problem requiring multi-level solutions. Surveillance must integrate parasites and pathogens; molecular studies should be connected with organismal and colony-level outcomes; pesticide assessment should incorporate realistic mixtures, sublethal effects, and interspecific differences; and candidate treatments require ecological validation. Climate change adds urgency by altering phenology, host–parasite interactions, and the potential ranges of pests and diseases.

Future progress will depend on closer integration of molecular biology, epidemiology, toxicology, ecology, spatial monitoring, and practical beekeeping. Coordinated surveillance across countries, rapid diagnostic technologies, standardized sampling protocols, and predictive approaches incorporating climate and landscape information will become increasingly important. Protecting pollinators in a changing world will ultimately require not only treating individual diseases or controlling pests, but understanding and managing the interacting biological and environmental processes that determine pollinator resilience.
